# The War

**Published:** 1899-11-04

**Authors:** 


### The War.

Once more, in the course of her long and splendid history, Great Britain has been called upon to apply to a recalcitrant the last argument of kings, and to defend the liberties of her outlying subjects at the cost of war with adversaries who display the qualities of valour and resolution, and who are so far worthy of the steel against which they have ventured to contend. Upon such a contest the eyes of the whole nation will be rivetted, not with any question as to the justice of the cause, or the ultimate results of the struggle, but only with anxiety for the fate of brave men, and with sympathy and sorrow for those who will be called upon to mourn for the loss of their best beloved. There is no section of the community, outside of the actual fighting line, upon which the casualties of war fall so heavily as upon the doctors; a fact sufficiently illustrated, if illustration were required, by the proportion of their Victoria Crosses to those won by the other branches of the service. Already, and as a consequence of the preliminary and partial engagements by which the outnumbered forces in Natal have been delaying the advance of the enemy pending the arrival of adequate reinforcements from England, officers of the medical department of the Army have been left in charge of the wounded at Dundee, to become prisoners in the hands of the Transvaal Boers; a lot which promises to combine, with every description of military discomfort, a complete absence of the compensating advantages which are afforded by participation in the triumphs as well as in the hardships of a campaign, and by the power of bringing much needed help to warm friends and trusted comrades.

There is a striking passage in one of Von Moltke's letters to Professor Bluntschli, in which he says that peace is a dream, and not even a beautiful one, war being manifestly, to those who will put away the veil of sentiment, and will look facts in the face, a definite element in the providential government of the world, and being the source, or at least the nursery, of some of the highest of human virtues. Those who are old enough to remember the social state and tendencies of this country before the Crimean War, and the change which that war produced in many departments of thought and feeling, will be but little disposed to question the accuracy of Von Moltke's view; a view powerfully maintained in the stirring verses which were contributed to the *Times* of Monday last by the Archbishop of Armagh. At the close of the first half of the century some of the better aspects of the national character were well nigh submerged beneath the advancing wave of luxury, and patriotism was in danger of being choked by what the Apostle described as covetousness. We are indebted to the war with Russia for the Volunteers, and for all which their existence signifies, including, among many other things, that growth of the Imperial spirit, both at home and in the colonies, which has rallied our Australian and Canadian brethren round the flag of England, and has made every English heart swell with pride at the eagerness with which they have come forward to assist in defending, for their fellow-subjects in the Transvaal, the liberties which have been so freely accorded to themselves. We venture to think that the war in which we are now engaged has been sufficiently "deplored," and that the time has come for recognising at once its justice and its necessity. If there be anything to deplore, it is that our enemies should have been suffered to strengthen themselves by elaborate preparations, by the construction of forts, and by the importation of arms and ammunition, for which they, as the people of a subject state, could have no possible occasion, and which could only have been directed against ourselves. England would have been within her rights in forbidding all this, and would probably have done so long ago if she had been governed by men who remembered that the business of rulers is to rule, and that statesmen should lead public opinion, instead of waiting to be guided by it. But, on the whole, there is not much of which we need complain. The delay in effecting the inevitable settlement will probably have the effect of rendering it more complete; and we have now all realised that Boerdom is an anachronism to be swept away, rather than a system with which it may be possible to effect a compromise.

